# Switching performance assessment of gate-all-around InAs–Si vertical TFET with triple metal gate, a simulation study

**DOI:** 10.1186/s11671-023-03816-6

**Published:** 2023-03-10

**Authors:** Dariush Madadi, Saeed Mohammadi

**Affiliations:** 1 Department of Engineering Sciences, Faculty of Technology and Engineering, Semnan University, Semnan, Iran; 2 Department of Electrical and Computer Engineering, Semnan University, Semnan, 3513119111 Iran

**Keywords:** InAs–Si heterojunction, Gate-all-around, Vertical TFET, Subthreshold slope, Triple metal gate

## Abstract

This study presents a gate-all-around InAs–Si vertical tunnel field-effect transistor with a triple metal gate (VTG-TFET). We obtained improved switching characteristics for the proposed design because of the improved electrostatic control on the channel and the narrow bandgap source. It shows an *I*_on_ of 392 μA/μm, an *I*_off_ of 8.8 × 10^−17^ A/μm, an *I*_on_/*I*_off_ ratio of about 4.4 × 10^12^, and a minimum subthreshold slope of 9.3 mV/dec at *V*_*d*_ = 1 V. We also analyze the influence of the gate oxide and metal work functions on the transistor characteristics. A numerical device simulator, calibrated to the experimental data of a vertical InAs–Si gate all around TFET, is used to accurately predict different features of the device. Our simulations demonstrate that the proposed vertical TFET, as a fast-switching and very low power device, is a promising transistor for digital applications.

## Introduction

MOSFETs' scaling down to enhance the device performance and increment the integration density has been pursued for decades [1–3]. However, short-channel MOSFETs cannot meet low-power application requirements due to the non-scalable subthreshold slope (SS) at 25 °C. For solving this problem, TFETs have been introduced [4–9]. The band-to-band tunneling (BTBT) current mechanism in TFETs is based on controlling quantum tunneling across the barrier rather than modulating thermionic emission over a barrier as in conventional FETs. Despite sub-60 mV/dec subthreshold swing, large *I*_on_ to the *I*_off_ current value (*I*_on_/*I*_off_), and very low *I*_off_, low *I*_on*,*_ and ambipolar current are disadvantages of TFETs. Vertical TFETs (VTFETs) are proposed to resolve the shortcomings of conventional TFETs [10–15]. VTFETs benefit from tunneling all over the source-channel interface that enhances the tunneling current. Further improvement may be obtained by employing low-bandgap semiconductors like Ge, InAs, and SiGe in the source side [12, 16–22], and using heterojunction of III–V compounds at the source-channel interface [11, 23]. Although the techniques mentioned above have significantly enhanced the conventional TFETs' switching properties, but the obtained results are far away for real-world applications, particularly in terms of drive current. The other drawback is the complexity of the fabrication process of some of the proposed structures.

This paper proposes a gate-all-around InAs–Si vertical TFET with the Triple Metal Gate (TMG) and investigates its switching behavior by using a calibrated numerical device simulator. The main idea of our work is the enhancement of on-state current by employing a narrow bandgap source and improvement of the device electrostatics by using the triple metal gate technique. In the next section, we describe the device structure, study the device operation by using a calibrated simulation framework and investigate the switching characteristics. Finally, we compare the achievements made by our device against those of some recently developed TFETs and conclude the paper.

## Device architecture

**Fig. 1:**
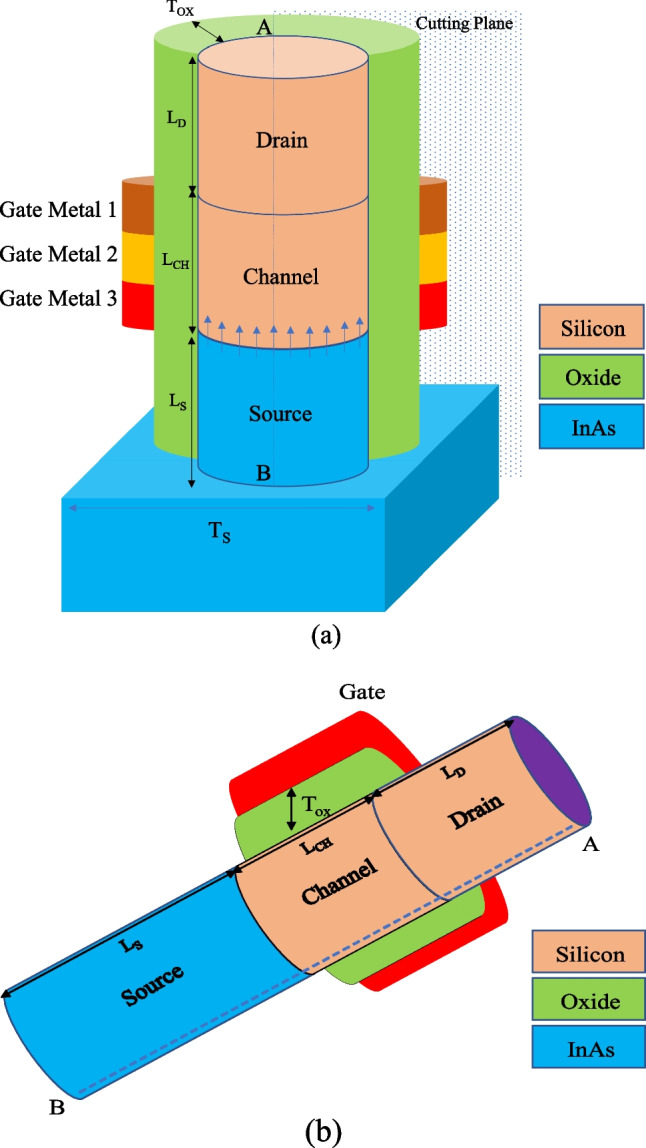
3-D view of the VTG-TFET (**a**) and GAA-TFET (**b**)

**Fig. 2:**
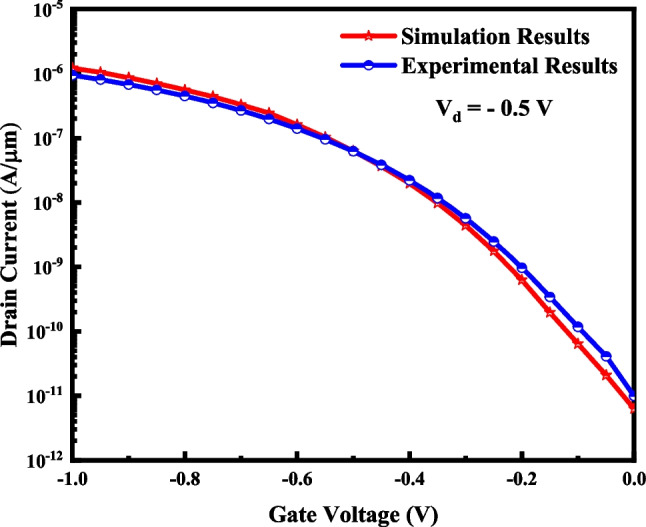
Calibration of the I–V characteristic of vertical InAs–Si TFET [25] and silvaco results

A 3-D view of the VTG-TFET is demonstrated in Fig. 1a. The device has an inverse T-shaped structure in which the InAs source region is located at the base, and silicon channel and drain sides are stacked on the source. The oxide region surrounds the Fin-shaped semiconductor region. Gate is symmetrically composed of three different metals, and their work functions are selected in a way that the best device performance is obtained. The channel length (*L*_CH_), the source length (*L*_S_), the drain length (*L*_D_), the channel thickness (*T*_CH_), and the dielectric thickness (*T*_OX_) are 50 nm, 70 nm, 30 nm, 10 nm, and 2 nm, respectively. It should be noted that HfO_2_ is employed as the gate oxide. The doping densities of the P-type InAs source area, the N-type silicon channel side, and the N-type silicon drain side are N_S_ = 5 × 10^19^ cm^−3^, N_CH_ = 5 × 10^15^ cm^−3^, and N_D_ = 8 × 10^18^ cm^−3^, respectively. In order to reduce the ambipolar current, the drain region is not heavily doped. Figure 1b illustrates an InAs–Si gate all around TFET (GAA-TFET) that we use as a reference structure. SILVACO TCAD [24] is utilized as a simulation framework to investigate the device's electrical characteristics. We initially calibrated the simulator by regenerating the experimental results of a vertical InAs–Si TFET [25] to validate our simulation results, as depicted in Fig. 2.

**Fig. 3:**
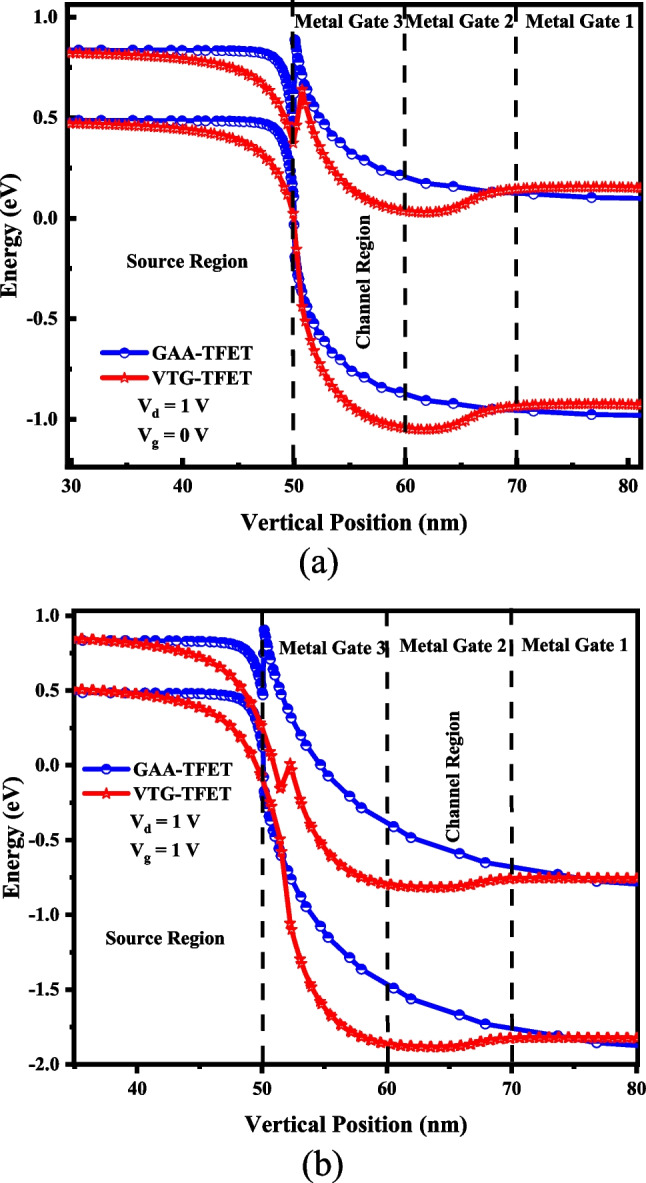
Both designs' energy band schematic in **a** OFF-state (*Vg* = 0 V, *Vd* = 1 V) and **b** ON-state (*Vg* = 1 V, *Vd* = 1 V) along the A–B cutline

Suitable physical models contain non-local BTBT, non-local trap-assisted tunneling (TAT), Shockley Read Hall (SRH) recombination, Fermi distribution, BGN, CVT, and drift–diffusion, are activated in the silvaco software. The effective tunneling masses of electron and hole are 0.322m_0_ and 0.549m_0_ for Si, and 0.026m_0_ and 0.57m_0_ for InAs, respectively [7, 26]. It should be noted that due to the large thickness of the Fin-shaped channel, the quantum confinement effects are not taken into account. The proposed and reference structures simulations consider two donor-like and acceptor-like traps with corresponding material-dependent parameters (shown in Table 1). As mentioned above, three metal gates are employed to modulate the energy bands and conduction of different channel parts and achieve the best switching performance of TFET. From the source side to the drain side, the work functions of the gate materials are set to 4.15 eV, 4.33 eV, and 4.15 eV, respectively. In Fig. 3, we show diagrams of energy bands for both devices in OFF-state (*V*_*g*_ = 0 V, *V*_*d*_ = 1 V) and ON-state (*V*_*g*_ = 1 V, *V*_*d*_ = 1 V) condition at the A–B cutline. As shown in the figures, using a narrow bandgap semiconductor in the source area expands the tunneling window across the junction in ON-state; moreover, the very low effective tunneling mass of charge carriers increases the BTBT tunneling rate. These features raise the *I*_on_ of the design structure. On another side, the OFF-state current may be considerably suppressed due to the straddling band alignment of the heterojunction and higher work function of the middle gate material.

**Table 1:** Simulation characteristic of donor-like and acceptor-like traps

Trap	Type	E. level (eV)	Density (cm^−3^)	Sign (cm^2^)	Sigp (cm^2^)	Deg
Si	Acceptor	0.56	1.0 × 10^13^	1.6 × 10^–16^	5.3 × 10^–15^	12
	Donor	0.56	8.6 × 10^12^	5.3 × 10^–15^	1.3 × 10^–14^	12
InAs	Acceptor	0.43	9.0 × 10^10^	5.6 × 10^–16^	2.4 × 10^–15^	12
	Donor	0.43	7.5 × 10^10^	1.2 × 10^–16^	8.8 × 10^–14^	12

**Fig. 4:**
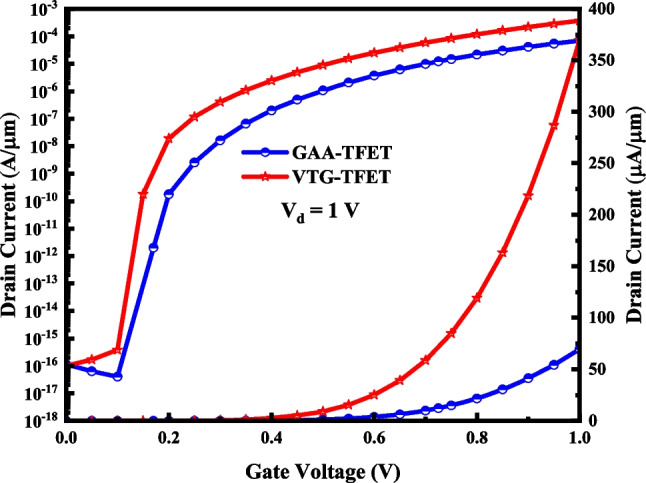
The I–V characteristic of VTG-TFET and GAA-TFET

A comparison among the transfer characteristics of VTG-TFET and that of reference structure is demonstrated in Fig. 4. We can understand from the figure that employing the triple metal gate (TMG) strategy leads to achieving a better electrostatic control on the channel and consequently lower *I*_off_, steeper SS, and higher ON-state current for the proposed structure. In Table 2, we evaluate the results of these TFETs quantitatively. As can be inferred from the table, we obtain a better I_ON_/I_OFF_ value in our VTG-TFET due to the lower *I*_off_ and enhancement of the *I*_on_ current.

**Table 2:** Quantitatively performance comparison of VTG-TFET and GAA-TFET structures

structures	*I*_ON_/*I*_OFF_	*I*_ON_ (A/µm)	*I*_OFF_ (A/µm)	SS_min_ (mV/dec)	V_TH_ (V)
TDG-VTFET	4.4 × 10^12^	3.92 × 10^–4^	8.8 × 10^–17^	9.3	0.61
GAA-TFET	6.04 × 10^11^	6.95 × 10^–5^	1.1 × 10^–16^	16.8	0.78

**Fig. 5:**
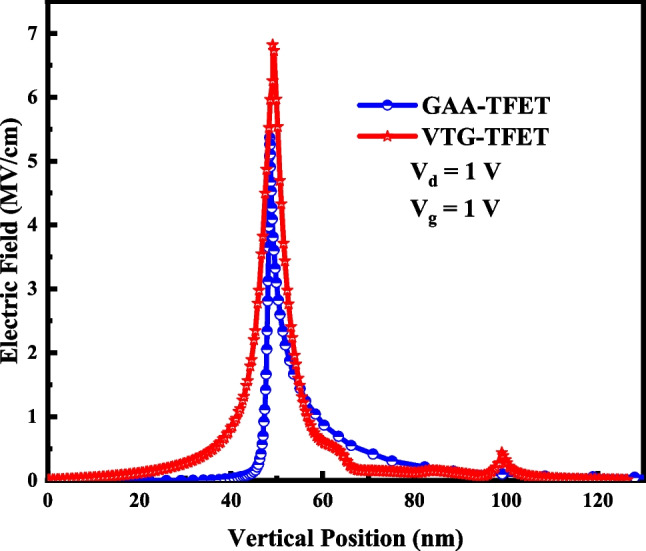
Lateral electric field of GAA-TFET and VTG-TFET at *V*_*d*_ = *V*_*g*_ = 1 V

**Fig. 6:**
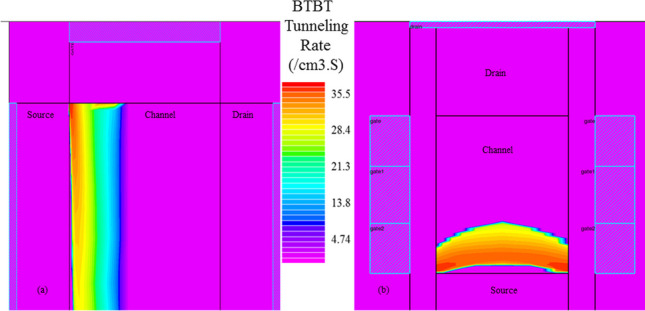
On-state BTBT Contour map of **a** GAA-TFET, and **b** VTG-TFET

The lateral electric field of VTG-TFET and GAA-TFET along the A–B cutline at *V*_*d*_ = *V*_*g*_ = 1 V is shown in Fig. 5. It is known that the BTBT generation rate depends on the electric field; hence, it is expected that the proposed structure with a higher peak of the electric field and a steeper gradient of the energy bands profile at the tunneling junction provides a larger ON-state current. The BTBT value of both structures at the same bias points is illustrated in Fig. 6.

**Fig. 7:**
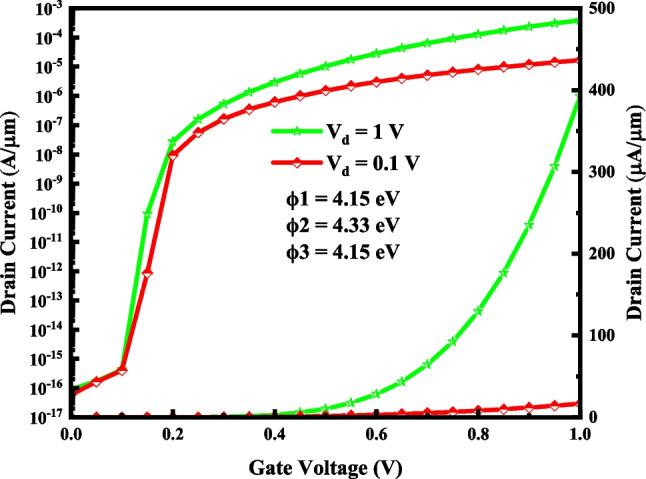
The I–V characteristic of VTG-TFET for two drain voltages

Figure 7 demonstrates the transfer characteristics of VTG-TFET at various drain voltages. As expected, the *I*_on_ increases by larger the drain voltage, while the *I*_off_ and subthreshold slope are almost constant. In the short channel devices, the effect of the drain voltage variation on the electrostatic of the channel and tunneling distance enhances, and as a result, the transfer characteristic of the structure changes more. The proposed TFET's switching behavior is also studied for various gate oxide materials.

**Fig. 8:**
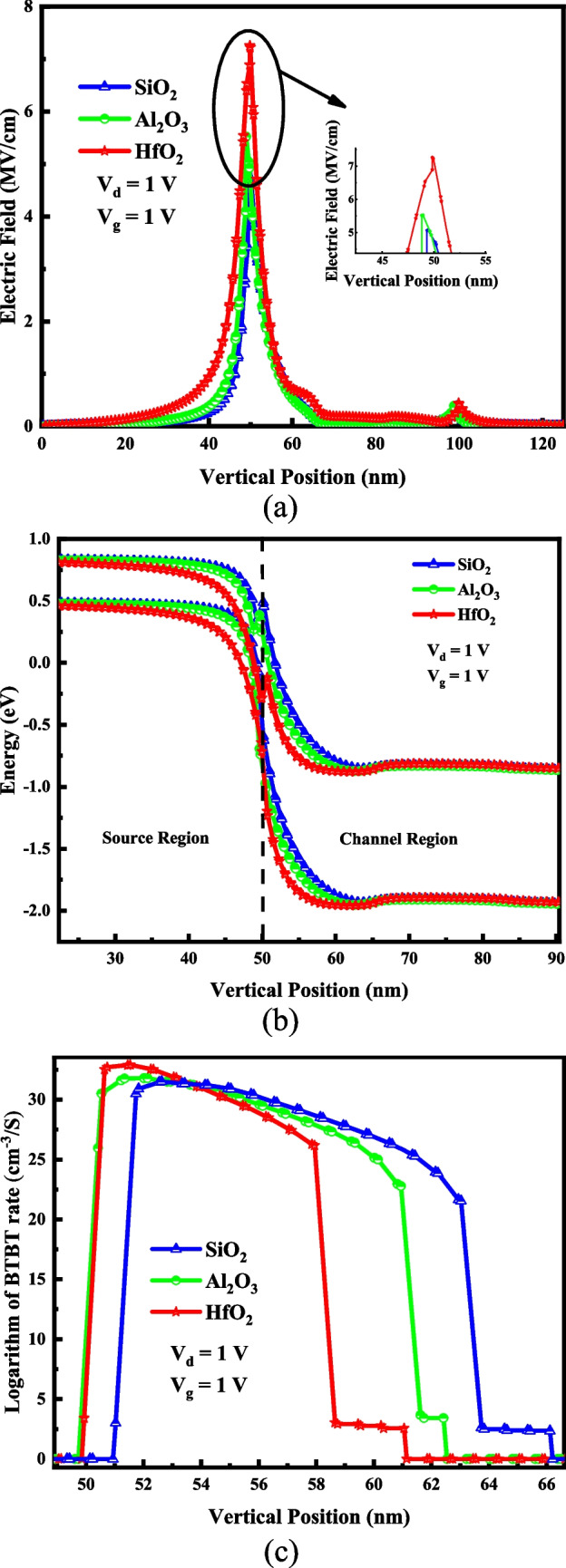

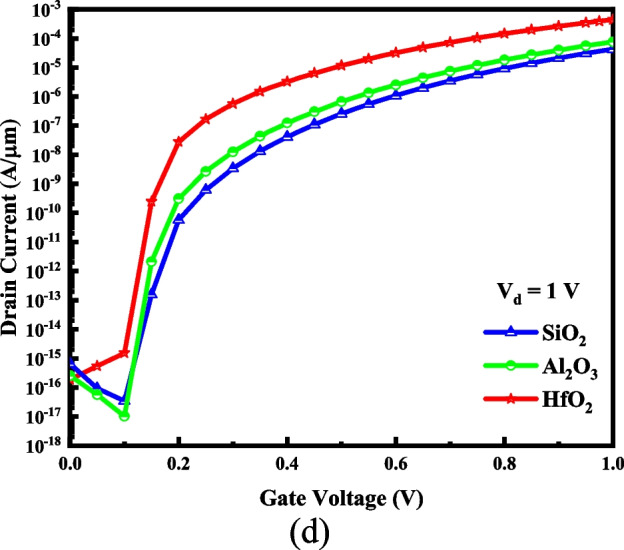
**a** Lateral electric field, **b** energy bands diagram, **c** BTBT generation rate, and **d** I–V characteristic of the VTG-TFET design for various gate oxide materials

**Fig. 9:**
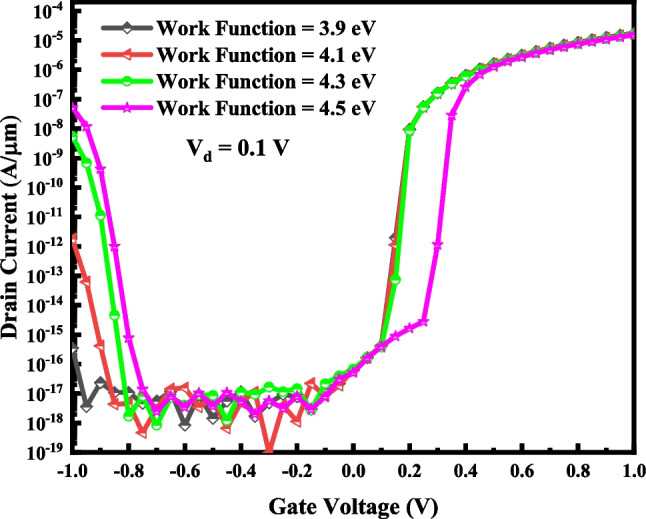
Effect of changing *ϕ*_1_ on the I–V characteristic of VTG-TFET with *ϕ*_2_ = 4.33 eV and *ϕ*_3_ = 4.15 eV

As shown in Fig. 8a, employing a dielectric material with higher-κ amplifies the electric field in the lateral position and at the tunneling interface. Furthermore, at the same applied voltages, it results in a smaller tunneling range (see Fig. 8b). Three different dielectric materials of HfO_2_ (*ɛ* = 25), Al_2_O_3_ (*ɛ* = 9), and SiO_2_ (*ɛ* = 3.9) are considered for the gate oxide. The enhanced electric field and shortened tunneling distance increase the BTBT generation rate for a device with higher-κ dielectric, as depicted in Fig. 8c. Figure 8d exhibits the I–V curve of VTG-TFET for three gate oxide materials. The steeper SS and higher *I*_on_ of the device with higher-κ dielectric are attributed to the increased electrostatic integrity of the channel and enhanced tunneling generation rate, respectively. In the multi-metal gate transistors, workfunction engineering to achieve the best device performance is necessary. Our device potentially has three metal gates with three various work functions. We name the workfunctions of the drain-side, middle and source-side gates, *ϕ*_1_, *ϕ*_2_, and *ϕ*_3_, respectively. Table 3 illustrates changing the *I*_on_, *I*_off_, I_on_/I_off_ value, and subthreshold slope of VTG-TFET for *ϕ*_2_ = 4.33 eV and *ϕ*_3_ = 4.15 eV, while *ϕ*_1_ changes. It is expected that the work function of the drain-side gate has no significant impact on the switching specification of the design. The I–V curve for various of *ϕ*_1_ is plotted in Fig. 9.

**Table 3:** Effects of changing *ϕ*_1_ on the switching characteristics of VTG-TFET with *ϕ*_2_ = 4.33 eV and *ϕ*_3_ = 4.15 eV

Work Function (eV)	I_ON_ (A/µm)	I_OFF_ (A/µm)	I_ON_/I_OFF_	SS (mV/dec)
3.9	1.75 × 10^–5^	6.24 × 10^–17^	2.81 × 10^11^	13
4.1	1.68 × 10^–5^	6.26 × 10^–17^	2.69 × 10^11^	12
4.3	1.61 × 10^–5^	6.8 × 10^–17^	2.37 × 10^11^	9.8
4.5	1.53 × 10^–5^	5.1 × 10^–17^	3 × 10^11^	11

The impact of the middle-gate workfunction is studied in Table 3. This gate can create a potential barrier at the middle of the channel that significantly blocks the leakage current and consequently reduces the off-state current.

**Fig. 10:**
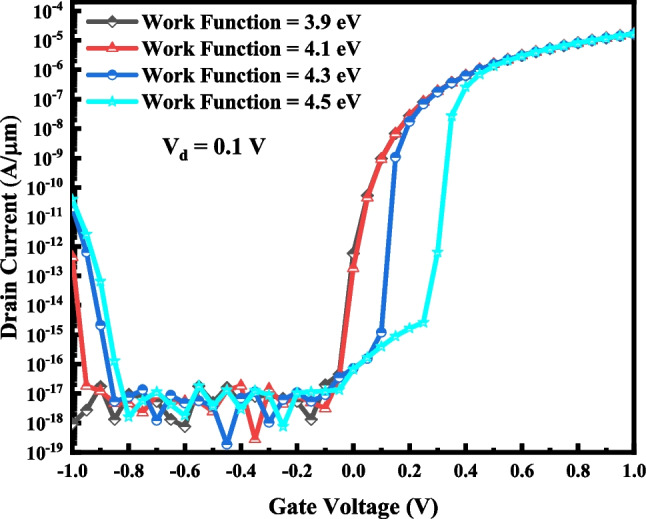
Effect of changing *ϕ*_2_ on the I–V characteristic of VTG-TFET with *ϕ*_1_ = 4.15 eV and *ϕ*_3_ = 4.15 eV

It should be noted that *ϕ*_2_ does not modulate the on-state current considerably, as shown in Fig. 10.

**Fig. 11:**
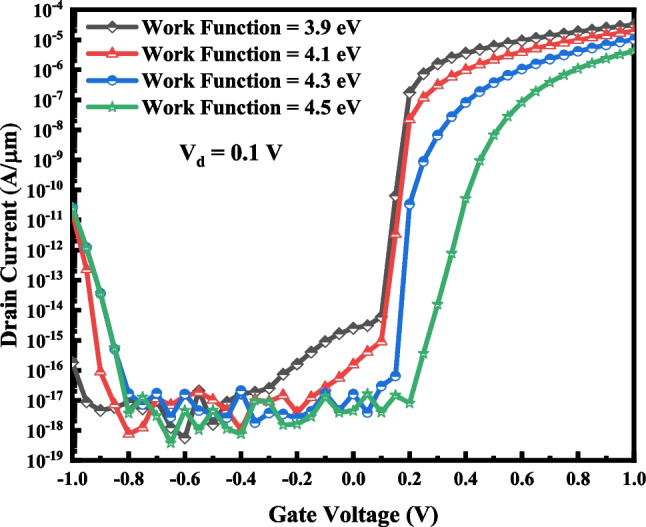
Impact of changing *ϕ*_3_ on the transfer characteristics of VTG-TFET with *ϕ*_1_ = 4.15 eV and *ϕ*_2_ = 4.33 eV

**Fig. 12:**
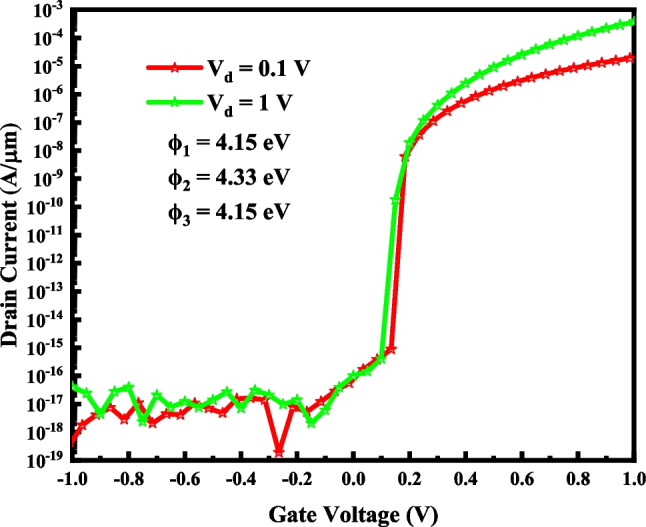
Ambipolar conduction is considerably diminished in VTG-TFET

The most influential work function on the switching performance of the device is that of source-side gate metal because of its vicinity to the tunneling junction. As depicted in Table 4, it can noticeably control the off-state current, on-state current and the steepness of the subthreshold slope. It is also illustrated in Fig. 11, where the I–V curve of VTG-TFET with various source-side gate metal work functions is exhibited. Ambipolar conduction is one of the main drawbacks of TFETs. In the case of VTG-TFET, we obtain the ambipolar characteristic of the design (as depicted in Fig. 12). It is anticipated that the ambipolarity is diminished noticeably due to the wide bandgap semiconductor employed in the channel-drain junction and different work functions of the gate metals.

**Table 4:** Effects of changing *ϕ*_3_ on the switching characteristics of VTG-TFET with *ϕ*_1_ = 4.15 eV and *ϕ*_2_ = 4.33 eV

Work Function (eV)	I_ON_ (A/µm)	I_OFF_ (A/µm)	I_ON_/I_OFF_	SS (mV/dec)
3.9	3.16 × 10^–5^	5.77 × 10^–13^	2.89 × 10^7^	12.2
4.1	1.93 × 10^–5^	1.81 × 10^–13^	9.19 × 10^7^	11.4
4.3	1.02 × 10^–5^	7.14 × 10^–17^	2.3 × 10^11^	8.4
4.5	4.31 × 10^–6^	6.60 × 10^–17^	2.5 × 10^11^	10.7

**Fig. 13:**
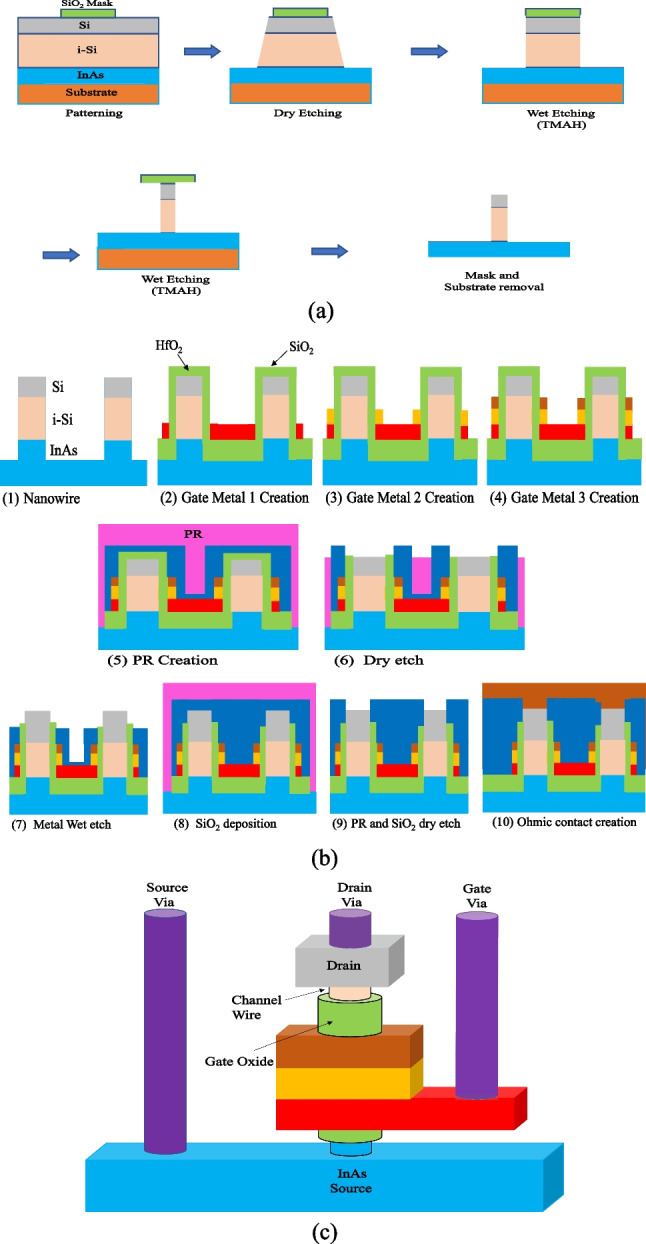
Illustration of **a** the fabrication process steps of the proposed TFET, and **b** complete structure of the VTG-TFET

For the sake of completeness, we propose a fabrication process flow to realize VTG-TFET, as depicted in Fig. 13. The fabrication methods of the vertical FETs are discussed in early studies [28–30]. Our proposed process starts with formation of a crystalline heterostructure of InAs/i-Si/Si (70/50/30 nm) using MOCVD on the silicon substrate. Then e-beam lithography is employed to create the SiO_2_ mask. TCP-RIE dry etching is carried out to form basic trapezoidal nanowire. The process is followed by anisotropic wet etching in TMAH acid to shrink the width of the nanowire. This method etches the nanowire layer under the oxide cover exclusively in the lateral direction that causes a very steep layer, as seen in Fig. 13a. The solution material helps to eliminate the plasma harm and even to clean the etched material surfaces. With such a technique, we can obtain the nanowire with a width of 10 nm. After removing the oxide mask, 2 nm ALD HfO_2_ gate oxide and the stack of metal gates are deposited (Fig. 13b2, b3, b4). Figure 13b-2 shows the metal 3 deposition. After that, we deposit the metal gate 2, and 1, and etched center between two FETs as shown Fig. 13b-3, and 4. Then 20 nm PECVD silicon dioxide were progressively deposited (Fig. 13b-5). The structure is covered with photoresist (PR), which facilitates eliminating the PR/SiO_2_/Metal/ HfO_2_ coats just at the top of the nanowire using dry etching (Fig. 13b-6). The oxide material surrounding the gate metals protects them from being etched, as seen in Fig. 13b-7. Additional oxide material is deposited and etched to enable formation of ohmic contacts (Fig. 13b-9). Finally, the metal electrodes have been created using rapid thermal annealing in N_2_ atmosphere. The complete architecture of the VTG-TFET (without isolation) is depicted in Fig. 13c.

**Fig. 14:**
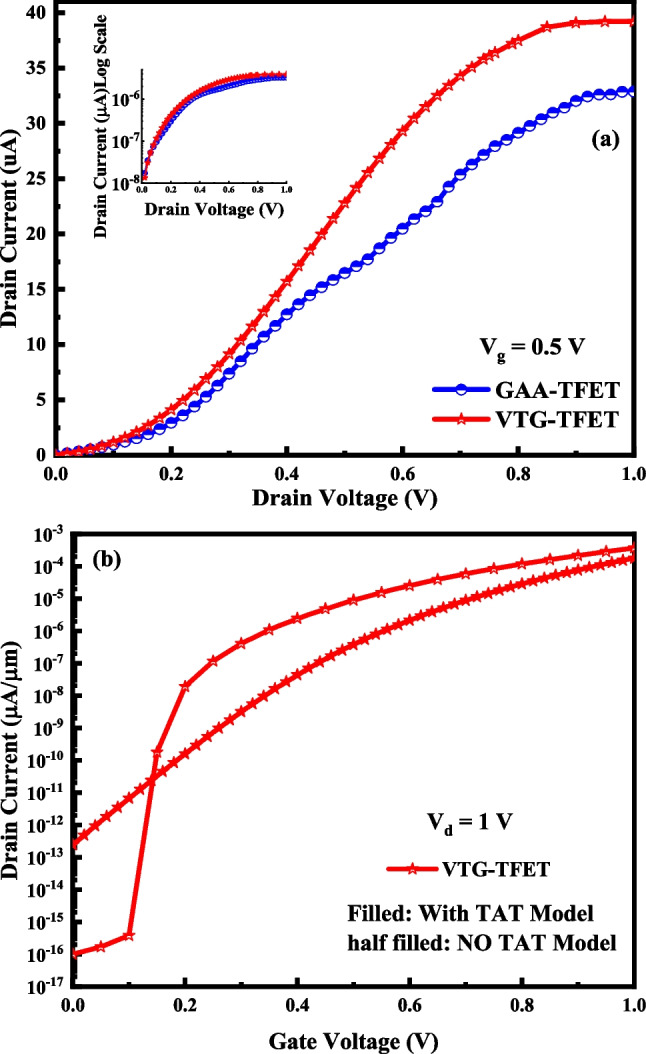
Illustration of **a** output characteristics for both devices and **b** I–V characteristic with consideration of TAT models

Achievements made by the proposed TFET are given in Tab. 5 and are analyzed versus the certain recently released TFETs. The provided data demonstrate that VTG-TFET has a great result in low operational voltage and is a reasonable alternative for low-power applications. Figure 14a demonstrates the output characteristics for both devices. It is depicted that; the results confirm the improvement of *I*_on_ in the proposed device. Figure 14b shows the impact of trap states on the transfer characteristics of VTG-TFET.

**Table 5:** Performance comparison of some recently proposed TFET structures versus the proposed device in this work

Reference	Exp/sim	*V*_*d*_ (V)	*V*_*g*_ (V)	SS (mV/dec)	*I*_ON_ (A/µm)	*I*_ON_/*I*_OFF_
[27]	Sim	1	1	~ 30	2 × 10^–5^	2 × 10^11^
[28]	Exp	0.1	1	~ 20	1 × 10^–8^	2 × 10^6^
[19]	Exp	− 0.5	0.5	~ 70	4 × 10^–6^	3 × 10^6^
[17]	Sim	1	1.5	~ 63	4.8 × 10^–8^	2.9 × 10^10^
[12]	Sim	0.5	1.5	~ 21.2	2.7 × 10^–5^	2.3 × 10^9^
[29]	Sim	− 1	− 1	~ 40	4.0 × 10^–4^	~ 1 × 10^6^
[30]	sim	− 1	− 1	~ 45	5.0 × 10^–6^	3 × 10^4^
[31]	sim	0.5	0.5	~ 21.6	1.2 × 10^–3^	5 × 10^9^
[32]	sim	0.2	0.4	~ 25	20 × 10^–6^	~ 1 × 10^11^
Our work	Sim	1	1	9.3	3.9 × 10^–4^	4.4 × 10^12^

**Fig. 15:**
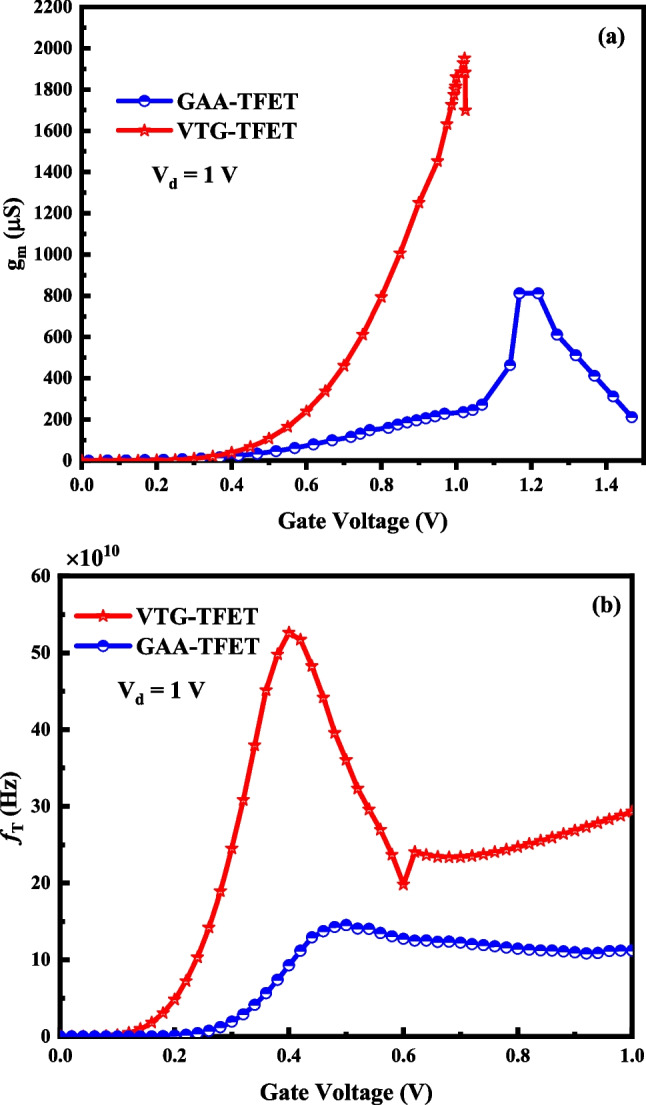
Illustration of *g*_*m*_ (**a**) and *f*_*T*_ (**b**) for both devices

In last section, we investigate the radio frequency (RF) characteristic for both devices. Figure 15a and b illustrates the transconductance (g_m_) and transition frequency (*f*_T_). The *f*_T_ of FETs is given by [33, 34]:

1 $$f_{T} = \frac{{g_{m} }}{{2\pi \left( {{\text{Parasitic}}\;{\text{Capacitance}}} \right)}}$$

As discussed, improvement of *I*_on_ in the proposed device leads the higher g_m._ The enhanced g_m_ and lower parasitic capacitance in the VTG-TFET leads the higher *f*_T_ which is depicted in Fig. 15b.

## Conclusion

A gate-all-around vertical TFET based on the InAs–Si heterojunction with a triple metal gate is proposed in this paper. Because of its excellent switching capabilities, our device is demonstrated to be an attractive design in low-power digital applications. An *I*_on_ of 392 μA/μm, an *I*_off_ of 8.8 × 10^−17^ A/μm, an *I*_on_/*I*_off_ current ratio of about 4.4 × 10^12^, and a minimum subthreshold slope (SS) of 9.3 mV/decade at 1 V operating voltage are obtained for VTG-TFET. The impact of gate metal work functions and gate dielectric materials are also investigated. To ensure the validation of the extracted data, the simulation software has been calibrated with the fabricated vertical InAs–Si gate-all-around TFET.

## Data Availability

The data that support the findings of this study are available from the corresponding author, upon reasonable request.
