# Correction: Switching performance assessment of gate-all-around InAs–Si vertical TFET with triple metal gate, a simulation study

**DOI:** 10.1186/s11671-024-03968-z

**Published:** 2024-02-12

**Authors:** Dariush Madadi, Saeed Mohammadi

**Affiliations:** 1 Department of Engineering Sciences, Faculty of Technology and Engineering, Semnan University, Semnan, Iran; 2 Department of Electrical and Computer Engineering, Semnan University, Semnan, 3513119111 Iran

**Correction: Discover Nano (2023) 18:37 ** 10.1186/s11671-023-03816-6

Following the publication of the original article [1], we were informed that:

a. citations [34] and [35] on p. 11 of the article PDF should be changed to [33] and [34]

b. respective references should be added to the reference list:

[33] 10.1109/ACCESS.2022.3228165

[34] 10.1007/s12633-023-02315-8

The original article has been corrected.
